# Comparison of Short-Term Clinical and Pathological Outcomes after Transanal versus Laparoscopic Total Mesorectal Excision for Low Anterior Rectal Resection Due to Rectal Cancer: A Systematic Review with Meta-Analysis

**DOI:** 10.3390/jcm7110448

**Published:** 2018-11-19

**Authors:** Mateusz Rubinkiewicz, Agata Czerwińska, Piotr Zarzycki, Piotr Małczak, Michał Nowakowski, Piotr Major, Andrzej Budzyński, Michał Pędziwiatr

**Affiliations:** 1 2nd Department of General Surgery, Jagiellonian University Medical College, 31-501 Kraków, Poland; mateusz.rubinkieiwcz@uj.edu.pl (M.R.); agata.czervinska@gmail.com (A.C.); piotr.zarzycki23@gmail.com (P.Z.); pmmalczak@gmail.com (P.M.); michal.nowakowski@uj.edu.pl (M.N.); piotr.major@uj.edu.pl (P.M.); andrzej.budzynski@uj.edu.pl (A.B.); 2 Centre for Research, Training and Innovation in Surgery (CERTAIN Surgery), 31-501 Kraków, Poland

**Keywords:** transanal, laparoscopic, rectal cancer, total mesorectal excision

## Abstract

Background: Transanal total mesorectal excision (TaTME) is a new technique that is designed to overcome the limits of the open and laparoscopic approach for rectal resections. Objective: This study is designed to compare TaTME with standard laparoscopic TME (LaTME). Methods: We searched Medline, Embase, and Scopus databases covering a up to October 2018. Inclusion criteria for study enrolment: (1) study comparing laparoscopic resection of rectal cancer vs. TaTME for rectal malignancy, (2) reporting of overall morbidity, operative time, or major complications. Results: Eleven non-randomized studies were eligible with a total of 778 patients. We found statistical significant differences in regard to major complications in favour of TaTME (RR = 0.55; 95% CI 0.31–0.97; *p* = 0.04). We did not found significant differences regarding overall complications intraoperative adverse effects, operative time, anastomotic leakage, intra-abdominal abscess occurrence, Surgical Site Infection, reoperations, Length of stay, completeness of mesorectal excision, R0 resection rate, number of harvested lymph nodes, circumferential resection margin, and distal resection margin. Conclusions: This meta-analysis shows benefits of TaTME technique regarding major postoperative complications. Regarding clinicopathological features transanal approach is not superior to LaTME. Currently, the quality of the evidence on benefits of TaTME is low due to lack of randomized controlled trials, which needs to be taken into consideration in further evaluation of the technique. Further evaluation of TaTME require conducting large randomized control trials.

## 1. Introduction

Many studies have confirmed that quality of surgery is an essential factor for good oncological outcome [1]. In the case of rectal surgery, this largely depends on the quality of mesorectum removal, which is often troublesome [2,3,4]. Middle and low portions of the rectum are the most challenging and technically demanding. The gold standard for rectal resections is yet to be established. Many hoped that laparoscopic surgery would significantly improve short- and long- term outcomes, but it was found to only be non-inferior in comparison to the open approach. For these reasons new techniques have been developed—Transanal total mesorectal excision (TaTME) being one of them [5,6]. TaTME is designed particularly to overcome the limits of the open and laparoscopic approaches, especially in obese, male patients with a narrow pelvis and very low rectal tumors. However, its oncological feasibility and safety is yet to be verified through large randomized control trials (RCTs). Although such studies have begun [7,8], the first results are only expected after 2020. Thus, a summary of current evidence is necessary to assess the evolution of this new technique. Currently, only a few studies comparing laparoscopic and TaTME approaches are available. Moreover, some of them, investigated in past meta-analyses, included data from abdominoperineal resections and even Hartmann procedures, which may bias the outcomes. Therefore, we designed an up-to-date meta-analysis of studies comparing the pure standard laparoscopic TME (LaTME) and TaTME procedures.

## 2. Methodology

### 2.1. Search Strategy

In October 2018, two teams of three researchers each, conducted a search of Medline, Embase, and Scopus databases for papers that had been published up to that time. To be as comprehensive as possible, the search had no language restrictions. The full search strategy for the OVID^®^ platform is available in Supplementary (Figure S1). Reference lists of relevant publications were assessed for additional studies and references from other systematic reviews or meta-analyses on the subject were hand-searched.

Inclusion criteria for study enrollment were studies (1) comparing laparoscopic resection of rectal cancer vs. TaTME for rectal malignancy, and (2) reporting overall morbidity, operative time, or major complications. Studies were excluded if they were not full-text papers or the extraction of mentioned outcomes was not possible. All teams identified and selected citations from the search independently. In cases of doubt about inclusion an attempt was made to reach consensus within the group. If no resolution was possible, a decision was made by the third reviewer. Data from included studies were extracted independently by all teams. Study quality was assessed using the Newcastle-Ottawa Scale [9].

### 2.2. Outcome Measures

The outcome measures of this systematic review were overall morbidity, intraoperative adverse events, operative time, anastomotic leakage, intra-abdominal abscess, reoperations, length of hospital stay (LOS), oncological outcomes, such as completeness of mesorectal excision, R0 resection rate, lymph node yield, distal resection margin (DRM), and circumferential resection margin (CRM).

### 2.3. Statistical Analysis

Analysis was performed using RevMan (Version 5.3, freeware from the Cochrane Collaboration). Statistical heterogeneity and inconsistency were measured using Cochran’s Q tests and I^2^, respectively. Qualitative outcomes from individual studies were analyzed to assess individual and pooled risk ratios (RR) with pertinent 95% confidence intervals (CI) favoring TaTME over the laparoscopic approach and by means of the Mantel-Haenszel random-effects method. When studies included medians and interquartile ranges, we calculated the mean ± standard deviation (SD) using a method that was proposed by Hozo et al. [10]. Weighted mean differences (WMD) with 95% CI are presented for quantitative variables using the inverse variance random-effects method. WMD and RR are presented on the graphs as squares, and overall WMD and RR is presented as diamond. Statistical significance was observed at a two-tailed 0.05 level for the hypothesis and with 0.10 for heterogeneity testing, while unadjusted *p*-values were reported accordingly. This study was performed according to the Preferred Reporting Items for Systematic reviews (PRISMA) guidelines and Meta-Analysis of Observational Studies in Epidemiology (MOOSE) consensus statement [11,12].

## 3. Results

**Figure 1:**
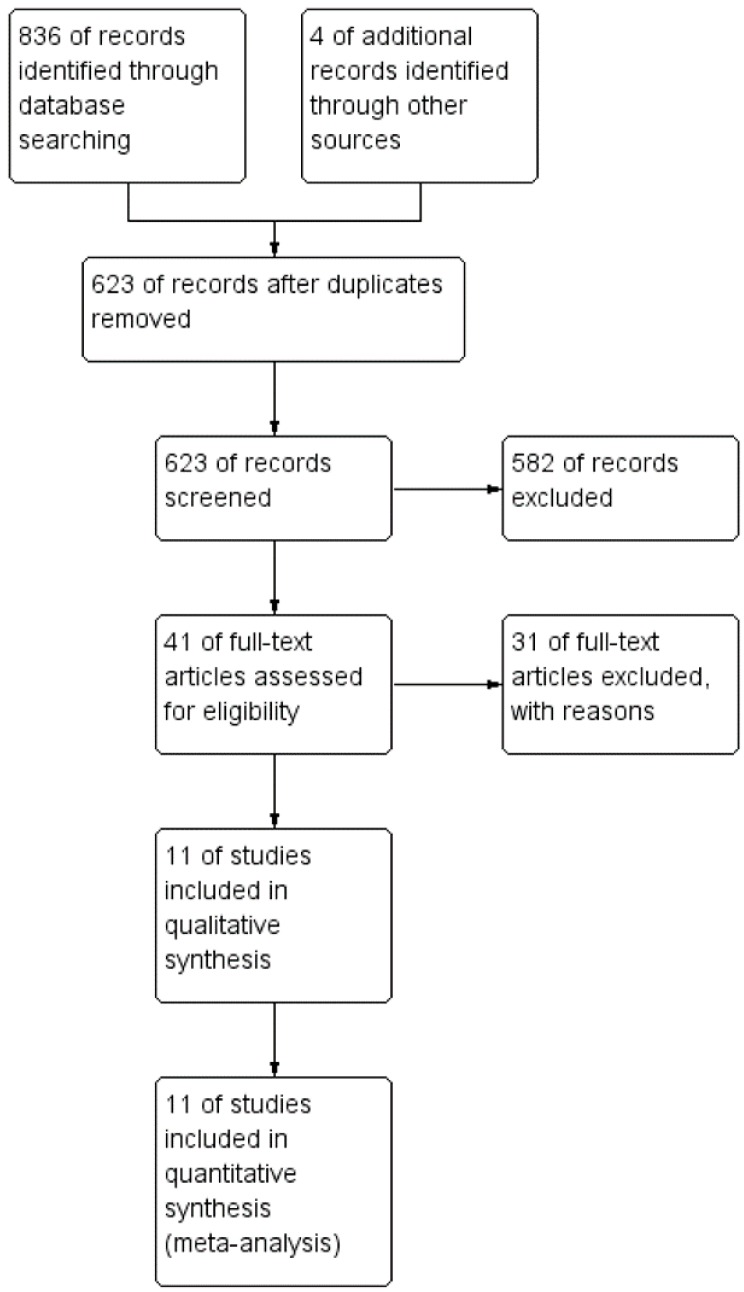
Preferred Reporting Items for Systematic reviews (PRISMA) Flowchart.

Using the strategy presented, our initial search yielded 836 records. A further four records were acquired through reference lists of relevant publications. After removal of duplicates, 623 references were screened through titles and abstracts resulting in 40 records, which were scheduled for full-text reading. In the end, we identified 11 studies that were eligible for inclusion and data extraction, covering 358 patients in the TaTME group and 420 patients in the laparoscopic group (778 patients in total). A PRISMA flowchart presented in Figure 1 summarizes the process used to obtain these studies. Baseline information on included studies is presented in Table 1. The quality of references, according to Newcastle-Ottawa Scale (NOS), is high.

**Figure 2:**
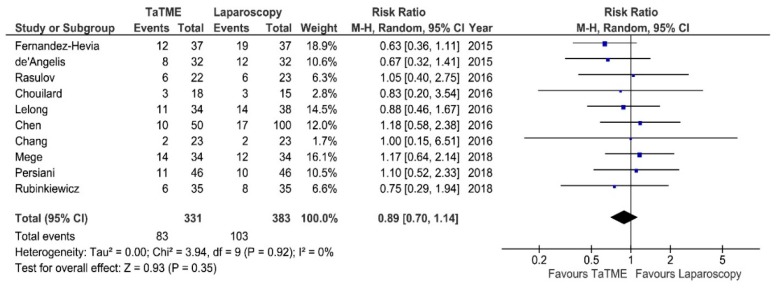
Overall morbidity. CI—Confidence Interval; df—degrees of freedom, M-H—Mantel-Haenszel test. WMD and RR are presented on the graphs as squares, and overall WMD and RR is presented as diamond.

**Figure 3:**
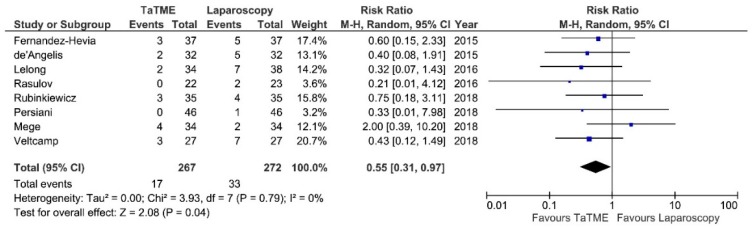
Major complications (Clavien-Dindo III–V). CI—Confidence Interval; df—degrees of freedom, M-H—Mantel-Haenszel test. WMD and RR are presented on the graphs as squares, and overall WMD and RR is presented as diamond.

Post-operative morbidity was reported in 10 studies. Meta-analysis revealed no statistically significant differences between analyzed groups: 83/331 (25%) in TaTME vs. 103/383 (27%) in Laparoscopy group: RR = 0.89; 95% CI 0.7–1.14; *p* = 0.35 (Figure 2). There was no heterogeneity among studies, I^2^ = 0%. Major complications (Clavien Dindo ≥3) were present in eight studies [24]. Meta-analysis revealed a statistically significant difference in favor of TaTME: 17/267 (6.36%) vs. 33/272 (12.13%): RR = 0.55; 95% CI 0.31–0.97; *p* = 0.04 (Figure 3). There was no heterogeneity among analyzed studies, I^2^ = 0%.

**Figure 4:**
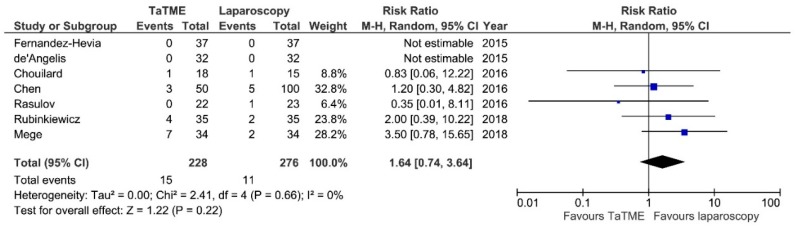
Intraoperative Adverse Effects. CI—Confidence Interval; df—degrees of freedom, M-H—Mantel-Haenszel test. WMD and RR are presented on the graphs as squares, and overall WMD and RR is presented as diamond.

Intraoperative adverse effects (IAE) were reported in seven studies. Meta-analysis showed no statistically significant differences between analyzed groups: 15/228 (6.57%) in TaTME vs. 11/276 (3.98%) in Laparoscopy group: RR = 1.64; 95% CI 0.74–3.64; *p* = 0.22 (Figure 4). There was no heterogeneity among studies, I^2^ = 0%.

**Figure 5:**
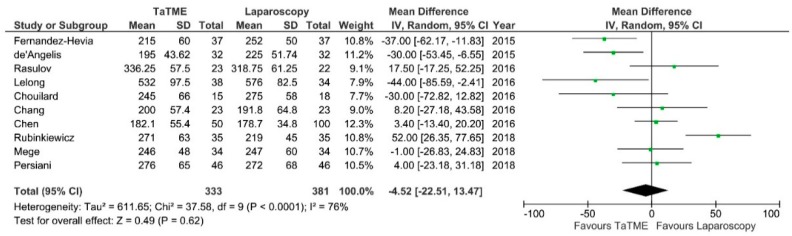
Operative time. CI—Confidence Interval; df—degrees of freedom, M-H—Mantel-Haenszel test. WMD and RR are presented on the graphs as squares, and overall WMD and RR is presented as diamond.

Operative time was reported by 11 authors. There were no significant differences between groups, however Fernandez-Hevia et al., De’Angelis, Rubinkiewicz et al. and Lelong et al. showed shorter duration of TaTME in comparison to laparoscopy: MD = −4,52; 95% CI −22.51–13.57; *p* = 0.62 (Figure 5). Only Fernandez-Hevia et al. report a two-team approach as standard; Chen, Rasulov et al., Persiani et al. and Veltcamp et al. used both a one- and two-team approach in their material. De’Angelis et al., Chang et al., Rubinkiewicz et al. and Mege et al. use a one-team approach. Lelong et al. and Chouilard et al. did not include information about the technique used. The heterogeneity between studies was significant, I^2^ = 76%.

**Figure 6:**
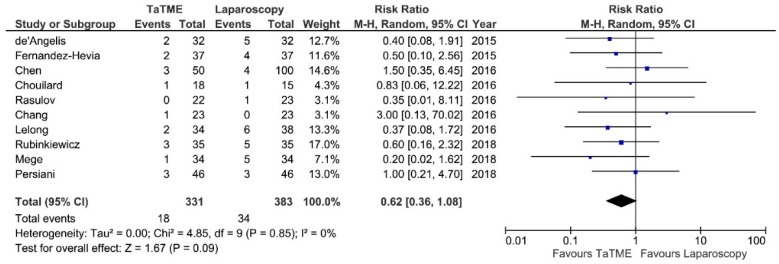
Anastomotic leakage. CI—Confidence Interval; df—degrees of freedom, M-H—Mantel-Haenszel test. WMD and RR are presented on the graphs as squares, and overall WMD and RR is presented as diamond.

Anastomotic leakage was reported in 10 studies. There were no significant differences between groups, 18/331 (5.43%) vs. 34/383 (8.87%): RR = 0.62; 95% CI 0.36–1.08; *p* = 0.09 (Figure 6). There was no heterogeneity among studies, I^2^ = 0%. We did not find any significant difference in intra-abdominal abscess occurrence (*p* = 0.51), Surgical Site Infection (SSI) (*p* = 0.98), reoperations (*p* = 0.81), and LOS (*p* = 0.11).

**Figure 7:**
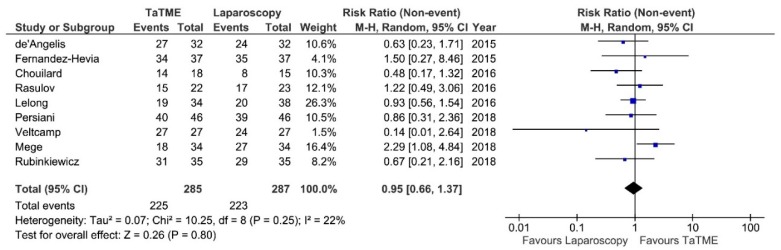
Completeness of mesorectal excision. CI—Confidence Interval; df—degrees of freedom, M-H—Mantel-Haenszel test. WMD and RR are presented on the graphs as squares, and overall WMD and RR is presented as diamond.

Completeness of mesorectal excision was reported in 9 studies. There were no significant differences between analysed groups: RR = 0.95; 95% CI 0.66–1.37; *p* = 0.80 (Figure 7). The heterogeneity of studies was moderate, I^2^ = 22%. All authors who report the quality of mesorectal excision used the Quirke classification with the exception of Fernandez-Hevia et al. and Mege et al., who did not state the protocol they used.

**Figure 8:**
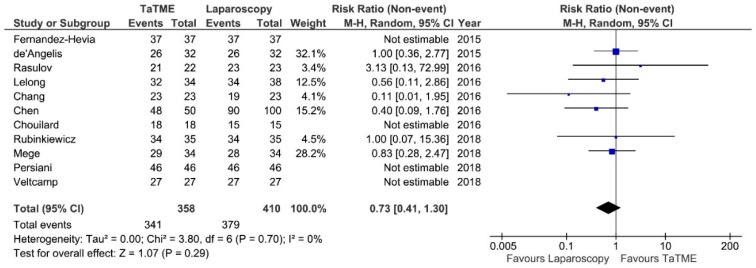
R0 resection rate. CI—Confidence Interval; df—degrees of freedom, M-H—Mantel-Haenszel test. WMD and RR are presented on the graphs as squares, and overall WMD and RR is presented as diamond.

R0 resection rate was reported by 11 authors. Meta-analysis revealed no significant differences: RR = 0.73; 95% CI 0.41–1.30; *p* = 0.29 (Figure 8). There was no heterogeneity between studies, I^2^ = 0%.

**Figure 9:**
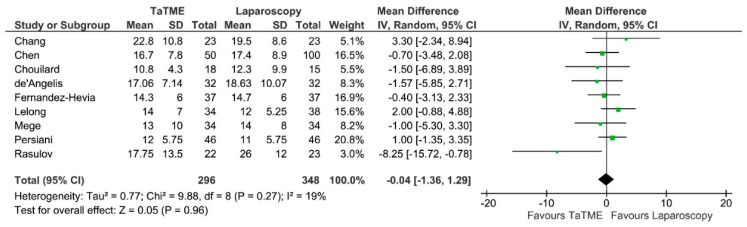
Number of harvested lymph nodes. CI—Confidence Interval; df—degrees of freedom; IV—inverse variance.

Number of harvested lymph nodes was provided in 9 studies. Analysis showed no difference between groups: MD = 0.04; 95% CI −1.36–1.29; *p* = 0.27 (Figure 9). There was no heterogeneity between studies.

**Figure 10:**
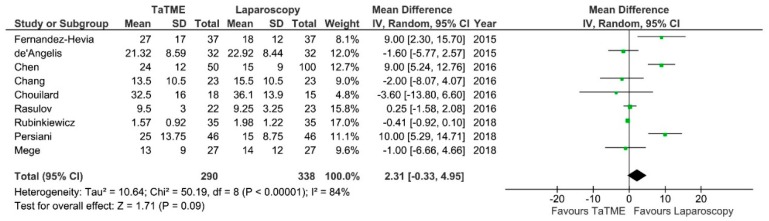
Length of distal resection margin. CI—Confidence Interval; df—degrees of freedom; IV—inverse variance. WMD and RR are presented on the graphs as squares, and overall WMD and RR is presented as diamond.

The length of DRM was present in nine studies. There were no significant variations among groups, however Fernandez-Hevia et al., Chen et al. and Persiani et al. reported greater distance in the TaTME group: MD = 2.31; 95% CI −0.33–4.95; *p* = 0.09 (Figure 10). Heterogeneity between studies is substantial, I^2^ = 82%. Rubinkiewicz et al. reported one case of positive DRM in the laparoscopy group.

**Figure 11:**
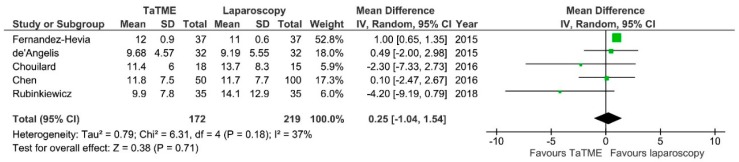
Circumferential resection margin. CI—Confidence Interval; df—degrees of freedom; IV—inverse variance. WMD and RR are presented on the graphs as squares, and overall WMD and RR is presented as diamond.

Five authors reported CRM. There was a significant difference between groups: laparoscopic procedures had a more narrow CRM: MD = 0.25; 95% CI −1.04–1.51; *p* = 0.71 (Figure 11). There was moderate heterogeneity between studies, I^2^ = 37%.

## 4. Discussion

This systematic review and subsequent meta-analysis, which included a total of 778 patients, shows that TaTME has a significantly lower rate of major complications defined as Clavien-Dindo ≥3. There were no significant differences in other clinical outcomes. Our meta-analysis failed to prove that TaTME could be associated with good quality of TME as a main measurement in an evaluation of surgery quality. Current studies do not assess long-term observation, thus it is impossible to prove the superiority of any technique in our meta-analysis.

Optimal surgical treatment of rectal cancer is yet to be achieved. The first laparoscopic surgeries were conducted a long time ago, but this approach is still considered as the gold standard of treatment, even though large randomized studies have failed to prove the oncological superiority of the laparoscopic approach. Meta-analysis of short- and long-term oncological outcomes showed no difference between laparoscopic and open approaches [1]. Furthermore, recent studies showed that there may be a smaller percentage of complete mesorectal excisions after the laparoscopic technique. However, patients themselves often prefer a minimally invasive approach, therefore its use will continue to increase [25,26,27]. TaTME is a novel technique aimed at better oncological outcomes. It is currently being thoroughly investigated by many researchers, resulting in many studies, none of which has been an RCT to date. There are meta-analyses published in the last three years, however they contain substantial bias, and a few of the more recently published studies are not included. Our meta-analysis, in contrast, included the several most recent papers (which were not included in previous analyses) and considered only pure TaTME and LaTME procedures [28,29]. Previous meta-analyses regarding feasibility of TaTME included abdomino-perineal resection (APR) or Hartmann resections, which in our opinion may have biased the results, rendering them inconclusive, especially when analyzing postoperative morbidity and anastomotic leakage [28,30,31]. Thus, we decided to exclude the studies by Perdawood et al., Koedam et al., Velthuis et al., and Marks et al. [32,33,34,35]. Zhang and Hu in their meta-analyses also included a randomized control trial by Denost et al., treating it as TaTME [28,31]. However, Denost et al. investigated intersphincteric resections from perineal approach, which in our opinion cannot be regarded as pure TaTME according to Sankt Gallen consensus [7,36,37]. What is also to consider, the study started in 2008, thus two years before the TaTME technique was introduced by its originator Prof. Antonio Lacy [38]. Similarly, we excludeed the study by Kanso et al., which for some unclear reasons was included in previous meta analyses [28,31,39]. In addition, a recently published meta-analysis by Hu et al. also included studies that compared laparoscopic TME with other transanal local excision [28,40]. Moreover, there are few statistical discrepancies in previously published meta-analyses which should be underlined. Zhang et al. reported better quality of CRM in TaTME group, however the statistical analysis is biased by incorrectly included study by Denost, which is responsible for 26% of weight of the whole analysis [31]. Also Jiang et al. reported wider CRM in TaTME group, nonetheless in their analysis a single study by Fernandez-Hevia responded for 93% of the whole effect [29]. Next, Hu et al. use fixed model of effects model in the pooled analysis, what is questionable due to heterogeneity of included studies [28]. All of these factors prompted us to conduct a new analysis, as free from bias as possible.

In our meta-analysis of TaTME, we proved that this new method is safe and comparable to laparoscopic surgery regarding overall morbidity. None of the included studies revealed significant differences in terms of intraoperative adverse effects, and no adverse effects or complications, such as purse string failure or problems with smoke evacuation were noted [41,42]. Overall morbidity also did not differ between groups. However, our meta-analysis showed a significant drop in major complications defined as Clavien-Dindo III–V in favor of TaTME. One of the factors affecting postoperative complications may be the length of surgery. Operative time did not differ between the groups. However, TaTME can be conducted in two ways: by one or two teams of surgeons [43]. A subgroup analysis considering one vs. two-team approaches would be the preferred course of action. Unfortunately, the majority of included studies did not define the exact method of TaTME. This is of great importance, since shorter operative time may decrease the overall stress on the body associated with the surgery. The heterogeneity in this parameter is moderate (I^2^ = 55%), which prompted us to pool data and analyze operative time in total, when considering that we could not establish which studies involved one- or two-teams. Chen reports significantly shorter operative time by using a two-team approach [17]. Lacy also revealed reduced operative time in comparison to other authors, however, that study was single armed, not comparative [44]. Shorter surgery is beneficial for patients, although a two-team approach requires more highly skilled laparoscopic surgeons and more equipment, which may limit the introduction of the TaTME technique in smaller centers. Besides, the difference of approximately 60 min seems to not be clinically relevant, considering that the most important aspect of the surgery is achieving the best possible quality of the resection.

Currently, one of the leading topics under constant discussion is the quality of mesorectal excision. The most recent study showed an association between plane of mesorectal excision, long-term overall survival and recurrence rate [45]. In our study, there were no differences between TaTME and LaTME regarding the quality of the specimen. However, TaTME is a relatively novel technique, which requires progression along a learning curve, whereas LaTME is an accepted, well investigated method of treatment that has been used for years. Koedam et al. underline that TaTME is a complex technique, which requires a minimum of 40 cases to reach acceptable perioperative outcomes [46]. The majority of available studies comprise small groups of patients, incorporating cases from the learning curve as well. Therefore, popularization of the TaTME technique may improve the outcomes in that field. Veltcamp et al. assessed residual mesorectum in patients post LaTME and TaTME using MRI. They found that almost 46% of patients had remnants of mesorectal tissue after LaTME. It should be noted that LaTME is considered as an independent risk factor for such a situation. Also, we found no differences between groups regarding DRM and R0 resection rate. In both techniques, margins are satisfactory. Moreover, nowadays as long as the margin is negative, the width of resection is irrelevant [45,47].

Our findings show, that TaTME is a promising technique, but its utility has to be further examined before it will be considered as an alternative to LaTME. In our opinion, the approach used should be based on the experience of a surgeon as well as the stage of cancer and the condition of a patient. Nowadays, a trend towards extending the indications for local excision in less advanced tumors with no adverse pathological risk-factors can be observed [48]. According to some authors, local resection may be feasible for the elderly, even up to T3 tumors with good long-term outcomes [49,50]. There are ongoing studies on non-operative management of rectal cancer [51,52]. However, the gold standard of treatment is still TME, which is also is also underlined in European Society for Medical Oncology (ESMO) guidelines [48].

Our study has some irrefutable limitations. The main limitation is the lack of RCTs to include in the analysis. These are still in progress and will be published in the next few years [7,8]. As mentioned above, the numbers of patients are small, which may influence statistical significance. The total number of patients included is still too small for adequate power of the study. Investigators of COLOR III studies calculated that at least 732 patients are required in the TaTME arm [7]. We also do not have full information about surgical proficiency of the investigators from studies included in the meta-analysis. In the included studies, patients were not randomized, which may create potential selection bias, especially at the learning curve stage, when surgeons prefer to operate easier cases. None of the authors apart from Rubinkiewicz et al. reported exclusion of initial cases due to the learning process, proved to be a learning curve of about 40 cases. Not all of the studies reported variables included to our meta-analysis. We did not contact the authors to achieve additional data, which were not published, although it would for sure improve the quality of final results. Next, the most important value of the new technique is possibly better oncological outcome, thus better overall (OS) and disease free survival (DFS). TaTME is a new approach, which requires more time for long-term observations. Currently, we lack data about OS and DFS. Thus, we need to wait until COLOR III and ETAP-GRECCAR have their final results, since long-term observations are included in the study designs. An important consideration is that two authors (Fernandez-Hevia and Mege) did not state whether they used the Quirke classification, a standard protocol in pathological assessment of surgical specimens [48,53], which creates a potential source of bias in our results.

Conclusion: This is an updated meta-analysis including the most recently published trials and comprising only studies comparing pure TaTME with LaTME. The analysis showed the benefits of the TaTME technique regarding major postoperative morbidity but not for CRM and DRM as suggested previously. In clinicopathological features, the transanal approach is not superior to LaTME. Currently, the quality of the evidence on benefits of TaTME is low due to lack of randomized controlled trials. To properly evaluate the feasibility, safety, and efficacy of TaTME, the results of large RCTs are required.

## Figures and Tables

**Table 1:** Summary of included studies.

First Author	Year	Country	Study Design	N TaTME/Lap	% Male TaTME/Lap	Age TaTME/Lap	Distance to Anal Verge TaTME/Lap [mm]	T0–T2/T3–T4 TaTME	T0–T2/T3–T4 Lap	NOS
Fernandez-Hevia [13]	2015	Spain	C	37/37	65/59	64.5/69.5	ND	8/28	10/24	9
de’Angelis [14]	2015	France	CM	32/32	66/66	64.91/67.16	4/3.7	13/19	16/16	8
Lelong [15]	2016	France	C	34/48	68/58	ND	ND	19/15	27/11	9
Chang [16]	2017	China	C	23/23	57/57	62.4/62.9	4.3/5.9	ND	ND	8
Chen [17]	2016	China	C	50/100	76/76	57.3/58.3	5.8/6.7	ND	ND	9
Rasulov [18]	2016	Russia	C	22/23	50/61	56/60	ND	5/17	7/16	9
Chouilard [19]	2016	France	C	18/15	33/47	55.4/57.8	<70/<70	10/8	8/7	9
Mege [20]	2018	France	CM	34/34	68/68	58/59	13/22	10/24	17/12	9
Persiani [21]	2018	Italy	CM	46/46	65/67	69/66.5	55/60	ND	ND	8
Veltcamp [22]	2018	Netherlands	C	27/27	67/74	68/67.2	ND	16/11	15/12	8
Rubinkiewicz [23]	2018	Poland	CM	35/35	35/35	64.3/60.3	29/31.9	21/14	24/11	9

C—cohort study; CM—case matched study; ND—no data; N—number of cases; % Male—percentage of male patients in groups; T0–T2/T3–T4—number of cases according to T stage in AJCC staging classification; NOS—Newcastle-Ottawa scale.

## Supplementary Materials

The following are available online at http://www.mdpi.com/2077-0383/7/11/448/s1, Figure S1: Searching strategy.
