# Supplementary figures and images for: Comparison of Short-Term Clinical and Pathological Outcomes after Transanal versus Laparoscopic Total Mesorectal Excision for Low Anterior Rectal Resection Due to Rectal Cancer: A Systematic Review with Meta-Analysis
Source: J Clin Med. 2018 Nov 19;7(11):448. doi: 10.3390/jcm7110448 (PMC6262322; doi:10.3390/jcm7110448)

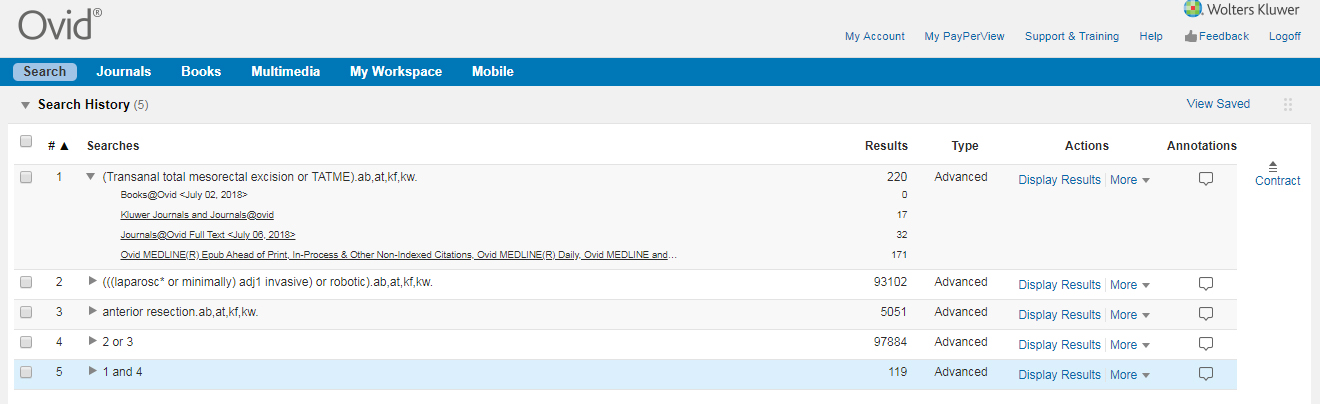

Supplement: Supplementary file 1 [file jcm-07-00448-s001.jpg]
